# Effects of cisplatin on olfactory function in cancer patients

**DOI:** 10.1038/sj.bjc.6602544

**Published:** 2005-04-12

**Authors:** A Yakirevitch, Y P Talmi, Y Baram, R Weitzen, M R Pfeffer

**Affiliations:** 1 Department of Otolaryngology-Head and Neck Surgery, The Chaim Sheba Medical Center, Tel-Hashomer 52621, Israel; 2 Oncology Institute, The Chaim Sheba Medical Center, Tel-Hashomer 52621, Israel; 3 Tel Aviv University, Sackler School of Medicine, Israel

**Keywords:** cisplatin, olfaction, smell

## Abstract

A prospective analysis of olfaction was performed in 21 patients receiving cisplatin. A reduction in olfactory function was noted in only one patient. Hearing impairment was documented in nine patients, none of whom had impaired sense of smell. We conclude that cisplatin has no major deleterious effect on olfactory function at doses which cause hearing impairment.

Disorders associated with a decreased taste sensation may result in a deleterious effect on quality of life and may contribute to reduced food intake and malnutrition, thereby increasing the side effects of cancer therapy (Comeau *et al*, 2001).

Cisplatin neurotoxicity (Peltier and Russell, 2002) and ototoxicity (Schaefer *et al*, 1985; Bartoshuk, 1995) are widely recognised, with hearing impairment reported in up to 81% of patients treated with high-dose cisplatin (Laurell and Jungnelius, 1990). Cisplatin has been reported to have a deleterious effect on taste sensation (Wickham *et al*, 1999). We therefore hypothesised that high-dose cisplatin might result in a reduced sense of smell. Olfactory cells are bipolar neurons that undergo constant regeneration with an average turnover time of about 30 days (Schiffman, 1997). The direct application of cisplatin to the olfactory apparatus in a guinea-pig model caused atrophy of the olfactory epithelium, olfactory nerve and olfactory bulb (Zhou and Lin, 1995). Several subjective reports have noted that up to 52% of patients receiving cisplatin were aware of smell changes (Rhodes *et al*, 1994; Wickham *et al*, 1999; de Graeff *et al*, 2000; Schiffman and Graham, 2000), but only one previous study has prospectively addressed the issue of olfactory alterations after chemotherapy (Ovesen *et al*, 1991). We therefore carried out a prospective study incorporating objective qualitative tests of olfaction in order to test our hypothesis that cisplatin will result in an impaired sense of smell in cancer patients. In addition, we investigated other factors that may influence olfactory function. These included disease status, smoking history and serum zinc levels. Auditory function tests were carried out as a control parameter for cisplatin toxicity.

## PATIENTS AND METHODS

Patients over 18 years of age admitted to the oncology department at the Chaim Sheba Medical Center for intravenous chemotherapy including cisplatin at a dose of at least 75 mg m^−2^ per cycle and who were planned to receive a cumulative dose of cisplatin greater than 200 mg m^−2^ were recruited to this study. Exclusion criteria included a history of chronic rhino-sinusitis or sino-nasal surgery, a tumour involving olfactory pathways or brain, and prior irradiation therapy to the head and neck region. The study was approved by the hospital ethics (Helsinki) committee and all participating patients gave written informed consent before inclusion in this study. All patients had normal blood count and renal function (24 h creatinine clearance >50 ml min^−1^) and received standard hydration (>2500 ml) and a standard intravenous antiemetic protocol (8 mg Ondansetron or 3 mg Granisetron together with 8 mg dexamethasone).

The study group comprised 21 consecutive patients (13 males and eight females) admitted between July 2003 and June 2004 for the treatment of a variety of primary tumours (upper digestive tract, lung, cervix, unknown primary carcinoma, malignant melanoma or metastatic seminoma). The patients were treated with standard departmental chemotherapy protocols for their tumour types, all of which included cisplatin in doses of 75 mg m^−2^ cycle^−1^ or more combined with either 5-fluorouracil, or with etoposide (VP-16) (with or without bleomycin), or with temozolomide according to tumour type. The patients' ages ranged from 28 to 78 years (mean 53.6 years). At enrolment, a detailed history was obtained, including demographic details, smoking history, tumour type and co-morbid pathology.

## METHODS

Olfactory function was tested before beginning chemotherapy (baseline), and after each course of cisplatin. The final olfactory assessment was performed at least 3 weeks after the last course of cisplatin.

Olfaction was assessed using the ‘Sniffin’ Sticks®' kit consisting of odorants in felt-tip pens (Wolfensberger *et al*, 2000). For odour presentation, the opened pens are positioned approximately 2 cm in front of both nostrils (all patients were tested bilaterally) for 3–4 s at intervals of 30 s. A patient has to choose one of four items that best describes the presented odour. The number of correctly chosen items gives the olfactory status score.

The sign test and Wilcoxon signed rank test for matched samples were used to estimate the statistical significance of the changes. The Spearman rank correlation was calculated for estimation of the relationship between the changes and the number of measurements.

Standard audiometry monitoring was performed. Serum zinc levels were measured in 14 patients.

## RESULTS

In all, 21 patients completed the planned chemotherapeutic treatment. They received one to six (mean 3.4) cycles of chemotherapy, with cumulative doses of cisplatin ranging from 180 to 945 mg and a mean cumulative dose of 525 mg (330 mg m^−2^). In one patient, a 60% decrease in olfaction was noted after the third course of treatment and a cumulative dose of cisplatin of 225 mg m^−2^. No decrease in olfactory function was found in 20 (95.3%) patients and in fact 10 patients had a final olfaction score that was higher than the pretreatment score. These score changes were statistically significant (*P*=0.0117 for the sign test and *P*=0.0322 for the Wilcoxon test). However, the rank correlation between the number of measurements and the improvement in olfactory function in these patients (ro=0.505, *P*=0.014) suggests that this may be a result of learning effect.

Hearing impairment was documented in nine patients (bilateral high tone sensori-neural hearing loss in all cases). The single patient whose olfaction decreased had no hearing loss.

Of 14 patients whose serum zinc levels were analysed, it was normal or increased in 12, according to normal ranges (75–120 mcg dl^−1^). In two patients reduced levels of zinc were seen after the second and third chemotherapeutic course, respectively. Neither of these patients had impaired olfaction. In one of them hearing impairment was registered. The patient whose olfactory function deteriorated had normal serum zinc levels.

## DISCUSSION

Cisplatin-induced olfactory deterioration is a possible consequence of the recognised neurotoxicity of this agent (Bartoshuk, 1995; Comeau *et al*, 2001; Peltier and Russell, 2002). An additional factor that could aggravate this effect is zinc deficiency, which has been found to be associated with taste dysfunction (Heyneman, 1996). The specific role of zinc in the control of taste and smell is unknown, but it is functionally involved at several levels of cellular organisation (Ripamonti *et al*, 1998). Cisplatin has been reported to increase urinary zinc excretion and reduce serum zinc levels (Sweeney *et al*, 1989). Oral zinc administration has been shown to hasten recovery of the sense of taste in patients undergoing radiotherapy for head and neck cancer (Ripamonti *et al*, 1998). It was therefore suggested that cisplatin might impair olfaction not only by its neurotoxic effect, but also indirectly via depletion of serum zinc levels.

Until recently, it was difficult to accurately measure olfactory status due to a lack of accurate and reproducible tools. ‘Sniffin’ sticks' is a noninvasive odour identification kit with odours of varying consistencies, which has been validated and used in several studies investigating the olfactory apparatus (Hummel *et al*, 2001) and has demonstrated deterioration in olfactory threshold in patients receiving radiotherapy for nasopharyngeal cancer (Ho *et al*, 2002).

Though subjective olfactory function impairment has been reported by a considerable number of cisplatin-treated patients (Rhodes *et al*, 1994; de Graeff *et al*, 2000), the only objective study thus far failed to show such an effect. Ovesen measured olfactory thresholds in 22 patients prior to and 2–3 months after treatment with several chemotherapeutic agents. They did not find statistically significant changes in olfaction although it should be noted that in this study the testing was performed with binary dilutions of a single odorant (Ovesen *et al*, 1991).

The results of our study performed with a more sophisticated test of olfactory function confirm the lack of an early effect of cisplatin on olfactory function. A decrease in olfaction was noted in one patient (4.7%) in our study. The cumulative dose of cisplatin given to this patient was relatively low and chemotherapy was discontinued after three cycles because of disease progression. Our results are in accordance with the results of Ovesen *et al*, who also failed to demonstrate impaired olfaction following treatment with cisplatin. A quality-of-life questionnaire-based study reported a subjective change in sense of smell in 26% of patients following high-dose chemotherapy and bone marrow transplantation, although more patients reported an increase in smell sensation rather than a decrease (Epstein *et al*, 2002).

In our study, the incidence of hearing impairment, an accepted indicator of cisplatin neurotoxicity, was 48%, which is comparable with results of similar studies (Blakley and Myers, 1993). The single patient whose olfactory status did deteriorate had no detectable hearing impairment. Serum zinc levels were monitored in 14 (67%) patients in the study. Only two (12%) of them showed a tendency toward hypozincaemia. The single patient with impaired olfaction had normal zinc levels after cisplatin treatment.

Our study has several limitations. It is a single-blind study and did not include a control group of patients who were not receiving cisplatin through the comparable period of the study. The ‘Sniffin’ Sticks®' kit used allows assessment of odour differentiation acuity rather than olfactory thresholds evaluation. Minimal and maximal limits of olfactory performance measured with this kit do not allow recording of further improvement of olfactory function in patients with maximal score or further deterioration in patients with minimal score. The fact that in 10 of 21 patients the final olfaction score was higher than the pretreatment score can possibly be explained by the patients learning the odours used in the test over time. The rank correlation between the number of measurements and the improvement in measured olfaction supports the contention that this may be a result of learning effect. Despite these limitations to our study, the lack of a decrease in olfactory function in all 10 patients who developed cisplatin-induced hearing reduction indicates that cisplatin has no significant deleterious effect on olfactory function.

## CONCLUSIONS

Based on several subjective reports, cisplatin has often been cited as a cause of altered olfactory perception. The result of our study and the single other prospective study reported (Ovesen *et al*, 1991) is that cisplatin has no measurable effect on the sense of smell. We conclude that there is no early negative effect of cisplatin on olfaction. Further, long-term studies are warranted in order to evaluate the delayed effects of cisplatin on olfaction.
